# Biocompatible Conductive Hydrogels: Applications in the Field of Biomedicine

**DOI:** 10.3390/ijms23094578

**Published:** 2022-04-21

**Authors:** Yang Hong, Zening Lin, Yun Yang, Tao Jiang, Jianzhong Shang, Zirong Luo

**Affiliations:** College of Intelligence Science and Technology, National University of Defense Technology, Changsha 410073, China; hongyang16@nudt.edu.cn (Y.H.); linzening@nudt.edu.cn (Z.L.); yangyun17@nudt.edu.cn (Y.Y.); jz_shang_nudt@163.com (J.S.)

**Keywords:** conductive hydrogels, preparation materials, synthetic techniques, biocompatibility, biomedical applications

## Abstract

The impact of COVID-19 has rendered medical technology an important factor to maintain social stability and economic increase, where biomedicine has experienced rapid development and played a crucial part in fighting off the pandemic. Conductive hydrogels (CHs) are three-dimensional (3D) structured gels with excellent electrical conductivity and biocompatibility, which are very suitable for biomedical applications. CHs can mimic innate tissue’s physical, chemical, and biological properties, which allows them to provide environmental conditions and structural stability for cell growth and serve as efficient delivery substrates for bioactive molecules. The customizability of CHs also allows additional functionality to be designed for different requirements in biomedical applications. This review introduces the basic functional characteristics and materials for preparing CHs and elaborates on their synthetic techniques. The development and applications of CHs in the field of biomedicine are highlighted, including regenerative medicine, artificial organs, biosensors, drug delivery systems, and some other application scenarios. Finally, this review discusses the future applications of CHs in the field of biomedicine. In summary, the current design and development of CHs extend their prospects for functioning as an intelligent and complex system in diverse biomedical applications.

## 1. Introduction

Hydrogels are biocompatible and hydrophilic 3D polymer structures in which the liquid part is water. They can be naturally derived, artificial or semi-synthetic. Naturally derived hydrogels are found in animals, plants, and ecological environments in nature, as well as in different structures of the human body such as mucus, cartilage, meniscus, tendon, vitreous, etc. [1]. With the help of the hydrophilic 3D cross-linked network structure, hydrogels can accommodate a large amount of water or other water-based liquids (e.g., cell nutrient solution, tissue culture solution) without disintegrating. With the unique performance characteristics and the advantages of biocompatibility, stretchability, transparency, etc., hydrogels are very suitable for biomedical applications [2,3,4].

However, many problems have been exposed in the decades of the practical application of traditional hydrogels in the field of biomedicine. For example, traditional hydrogels either lack stability and sufficient mechanical strength or are poor in biocompatibility [5]. The fragility of traditional hydrogels makes them difficult to handle. At the same time, traditional hydrogels are difficult to sterilize due to their sensitivity to general sterilization methods leading to deteriorating sterilization effects. Furthermore, the presence of cross-linking agents in hydrogels synthesized using chemical cross-linking techniques adds yet another threat of toxicity beyond bacteria [6]. The abovementioned problems make it necessary to adjust and improve the properties of hydrogels to better meet the needs of practical applications in the field.

In this context, researchers have been trying to design smart hydrogels that better fit the needs of applications by adjusting their physical and chemical properties. According to the different types of responses to external stimuli, smart responsive hydrogels can be divided into physically responsive hydrogels (temperature, magnetic field, pressure, electric field, etc.), chemically responsive hydrogels (pH, blood sugar, etc.), and biologically responsive hydrogels (antibodies, antigens, enzymes, etc.) [7]. These smart hydrogels can be synthesized from single or multiple polymers and can provide a variety of functions [8]. For example, they can be adapted for many biomedical applications by modifying their physicochemical properties (e.g., mechanical properties, rheology, pH stability) as well as their 3D structures and chemical and biological components [9]. This customized intelligent design enables parallelization of internal physiological responses and external stimulus responses, as well as special monitoring of other adjustable properties [10].

**Figure 1:**
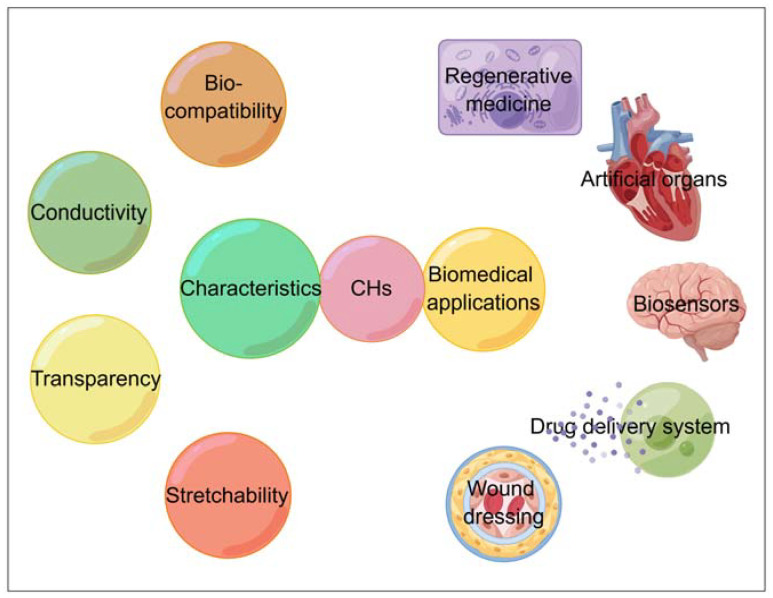
Characteristics and biomedical applications of CHs, by Figdraw (www.figdraw.com, accessed on 4 April 2022).

CHs are smart hydrogels with excellent electrical conductivity, belonging to the type of physically responsive hydrogels. However, the unique advantage of CHs is that their applications are not limited to excellent electrical conductivity, they can be designed to have some characteristics of other types of smart responsive hydrogels (temperature, pressure, glucose, etc.). As shown in Figure 1, with the continuous efforts of researchers, the physical and chemical properties of CHs, such as mechanical flexibility, electrical conductivity, self-healing, and biocompatibility, can be well regulated [11]. Hence, CHs have great application value in the field of biomedicine, such as regenerative medicine, biosensors, drug delivery systems, etc. [12]. Depending on the expected properties, CHs can be prepared based on physical or chemical interactions. The physical interactions include interactions between polyelectrolytes of different charges or between them and polyvalent surfactants/ions of different charges. While chemically developed CHs mainly depend on the covalent cross-linking of their polymer structures [1].

This review focuses on the development and applications of CHs in the field of biomedicine, especially in regenerative medicine, biosensors, drug delivery system, etc. In addition to the contemporary progress of CHs, this review also discusses the future cognition of their applications in the field of biomedicine.

## 2. Materials for Preparing CHs

The materials for preparing CHs are mainly divided into two parts according to their functions, one is the matrix materials that realize the basic properties of the hydrogels, and the other is the conductive materials that make them exhibit excellent electrical conductivity. Both the matrix materials and the conductive materials directly affect the physicochemical properties of the synthesized CHs. In this section, the types and properties of the two parts of materials used to prepare CHs are introduced in conjunction with Table 1. The classification of CHs in Table 1 is based on the types of conductive materials, matrix materials, and their applications, especially in the field of biomedicine.

### 2.1. Matrix Materials for Preparing CHs

The matrix materials, like the bricks that form the frame of a house, form a hydrogels matrix to which conductive materials can be added to give them conductive properties. Matrix materials are generally polymers with gel properties, either naturally occurring or artificially synthesized through the polymerization of monomers. Natural or synthetic polymers can be used separately, and mixing natural and synthetic polymers creates a unique hydrogel hybrid system [13].

Natural materials commonly used to construct hydrogels matrix include alginate (Alg.), gelatin (Gel), agar, chitosan (CS), etc., which are mostly cross-linked by hydrogen bonds. Synthetic materials commonly used to construct hydrogels matrix include acrylates (such as methyl acrylate, ethyl acrylate, 2-methyl methacrylate, 2-ethyl methacrylate), acrylic acid and its derivatives (such as polyacrylic acid (PAA), poly(methacrylic acid) (PMAA), poly(acrylamide) (PAM), poly(N-isopropyl acrylamide) (PNIPAM), vinyl sulfonate (such as sodium vinyl sulfonate, potassium vinyl sulfonate). There are also some other synthetic materials, such as hydroquinone, poly(ethylene glycol) (PEG), poly(dopamine) (PDA), poly(vinyl pyrrolidone) (PVP), and poly (vinyl alcohol) (PVA).

Natural hydrogels have great advantages in terms of degradability and biocompatibility, but they also have disadvantages such as poor material repeatability, low mechanical strength, and difficulty controlling structure and performance. In contrast, there are more types of synthetic hydrogels, and the use of synthetic methods to prepare hydrogels can effectively control their internal structures and properties so that synthetic hydrogels have properties and advantages that natural hydrogels do not have. And synthetic hydrogels have larger application space, but there are also problems such as poor degradability and biocompatibility [14,15]. At present, the shortcomings of natural hydrogels and synthetic hydrogels have been gradually repaired and improved under continuous research, and they now show better application effects.

**Table 1:** Typical examples of different types of CHs.

Types of CHs	Characteristics	Conductive Components	Materials	Conductive Hydrogel Polymer/System	Applications	Year	Refs
Electron-CHs (E-CHs)	Highly stretchable Better conductivity Better biocompatibility	Metallic nanoparticles	Ag	Polyacrylic acid (PAA)	Nanoelectronic devices Artificial muscles	2014	[16]
				Polyethylene glycol diacrylate (PEGDA)	Tissue engineering Chemistry reactions ware	2016	[17]
			Au	Poly(acrylamide) [poly (AAm)] and poly(N-isopropyl-acrylamide) [poly (NIPAAm)]	Chemoresistors Biosensors	2010	[18]
				Gelatin methacrylate (GelMA)	Cardiac tissue engineering	2016	[19]
			Cu	Polyacrylamide grafted poly(vinyl alcohol) (PAM-g-PVA)	Biosensors Drug delivery system	2008	[20]
		Carbon-based materials	CNTs (carbon nanotubes)	Gelatin-grafted-dopamine (GT-DA) Chitosan (CS)	Multifunctional bioactive dressings	2019	[21]
				N-isopropyl acrylamide (NIPAM)	Wearable electronics	2019	[22]
			Grphene /GO/rGO	Methacryloyl-substituted tropoelastin (MeTro)	Promising different biomedical applications	2016	[23]
				Poly(N-isopropylacrylamide) (PNIPAM)	Wearable electronics	2014	[24]
		Conducting polymers	Polyaniline (PANI)	Chitosan-graft-aniline tetramer (CS-AT) Dibenzaldehyde-terminated poly (ethylene glycol) (PEG-DA)	Drug delivery system	2016	[25]
				Poly(N-isopropylacrylamide) (PNIPAM)	Strain sensors Wearable electronics	2018	[26]
			Polypyrrole (PPy)	Polydopamine (PDA)	Wound dressing Biosensors	2018	[27]
			PEDOT: PSS	Iota-carrageenan (CRG) Polyvinyl alcohol (PVA)	Biomedical engineering Biomedical devices	2019	[28]
		Hybrid	Pt+ polyaniline	Pt nanoparticle (PtNP) Polyaniline (PANI)	Biosensors Biomedical devices	2015	[29]
			SWCNTs+ polyaniline	Single-walled CNTs (SWCNTs) Polyaniline (PANI)	Supercapacitor Wearable electronics	2018	[30]
Ion-CHs (I-CHs)	Highly stretchable Conductive Biocompatible Transparency Generate ionic gradients	Acids	H_2_SO_4_	Polyvinyl alcohol (PVA)-H_2_SO_4_ hydrogel Polyaniline (PANI)	Supercapacitor	2018	[31]
			H_3_PO_4_	Vinyl hybrid silica nanoparticles (VSNPs) Polyacrylamide (PAM)	Supercapacitor	2017	[32]
		Metallic salts	LiCl	Lithium chloride (LiCl) Acrylamide (AAm)	Soft actuators Electronic fish	2017	[33]
			Na^+^	Polyacrylamide (PAAm) Sodium chloride (NaCl)	Epidermal strain sensor Biosensors	2018	[34]
			Ca^2+^	Polyacrylamide-alginate (PAAm–alginate) Calcium chloride (CaCl_2_)	Stretchable ionic touch sensor	2018	[35]
			Al^3+^	Cellular-structured nanofibrous hydrogels (NFHs) Alginate	Biomedical engineering Biomedical devices	2017	[36]
			Fe^3+^	Polyethylene glycol/poly(acrylic acid) (PEG/PAA) double network hydrogel	Electronic skin Wearable electronics	2018	[37]
			K^+^	Polyacrylamide (PAAm)/carrageenan double network hydrogel	Thermistor	2018	[38]
		Ionic liquids	1-Ethyl-3-methylimidazolium chloride		Supercapacitor	2014	[39]
E-CHs and I-CHs	Highly stretchable Conductive Biocompatible Transparent/ semitransparent	Electron conductive components and ions	H_2_SO_4_^+^ PEDOT	PEDOT: PSS H_2_SO_4_	Wearable energy-storage devices	2017	[40]
			Na^+^ + Ca^2+^+ SWCNTs	Single-wall carbon nanotubes (SWCNTs) Sodium alginate Calcium chloride (CaCl_2_)	Wearable pressure sensor	2015	[41]
			Fe^3+^ + rGO	Poly(acrylic acid) (PAA) Polydopamine (PDA) Iron chloride (FeCl_3_)	Electronic skins Biosensors Tissue engineering	2018	[42]
			CuPcTs+ Polypyrrole	Crystal molecular copper-phthalocyanine-3,4′,4″,4‴-tetrasulfonic acid tetrasodium salt (CuPcTs)Polypyrrole(PPy)	Biomedical devices Biosensors	2015	[43]

### 2.2. Conductive Materials for Preparing CHs

According to the different conductive materials, CHs are mainly divided into electron-CHs (E-CHs) and ion-CHs (I-CHs). Different conductive materials or doping methods will affect the conductivity and other physical and chemical properties of CHs, which in turn affects their applications.

#### 2.2.1. E-CHs

The conductive materials of E-CHs are mainly divided into three categories: metal nanomaterials (Au, Ag, Cu, etc.), carbon-based materials (carbon grease, carbon nanoparticles/carbon nanotubes, graphene series, etc.), conductive polymers (polypyrrole, polyaniline, polythiophene, poly(3,4-ethylene dioxythiophene): poly(styrene sulfonate) (PEDOT: PSS, etc.) [44,45,46]. The easiest way to impart conductivity to hydrogels matrix is to incorporate and mix conductive materials. However, this poor mixing behavior results in poor electrical and mechanical properties of the prepared CHs [47]. To improve the performance of CHs, it has been found that the performance of CHs can be optimized by introducing special molecular interactions between conductive materials and polymer substrates, including non-covalent bonds (e.g., hydrogen bonds, electrostatic interactions, host-guest interactions) and covalent bonds (e.g., disulfide bonds, dynamic imine bonds, Diels-Alder reactions) [48,49,50,51,52].

Metal nanomaterials have the advantages of high electrical conductivity and high specific surface energy, so they are very suitable for preparing CHs. Gold nanomaterials are metal nanomaterials that are often used to prepare CHs. For example, photopolymerization of acrylamide or N-isopropyl acrylamide (NIPAM) in an aqueous solution containing gold nanoparticles can yield E-CHs with thermal responsiveness and tunable conductivity [18]. Zhao et al. [53] obtained E-CHs with thermal switching electronic properties by copolymerizing vinyl-functionalized gold nanoparticles with NIPAM. In addition to gold, copper nanomaterials are also commonly used to prepare CHs. Wei et al. [20] generated Cu nanoparticles in situ by reducing Cu^2+^ in the network of PAM grafted PVA, and the resulting CHs could be used as gas sensors. The introduction of metal nanomaterials into the hydrogels network has little effect on their mechanical properties, so such E-CHs are widely used in various stress-responsive sensors.

Carbon-based materials can build 3D conductive networks in polymer matrices to achieve conductive function. Moreover, such E-CHs have a high specific surface area and abundant surface functions, which can also improve the mechanical properties of the material [54]. Carbon-based materials mainly include carbon nanotubes, graphene and graphene oxide nanosheets, etc. The commonly used design method is to uniformly disperse these carbon-based materials in a hydrogels matrix and then construct 3D conductive networks through component cross-linking. Graphene and graphene oxide (GO) nanosheets are important materials for the fabrication of E-CHs. For example, Xu et al. [55] prepared functionalized graphene CHs suitable for supercapacitors by heating a mixture of GO and hydroquinone aqueous solution. In addition, Xiao et al. [56] utilized high-strength CHs fabricated from aqueous solutions of PVA, PEG, and GO, which were successfully applied to human electrocardiogram (ECG) electrodes.

In addition to metal nanomaterials and carbon-based materials, the unique conjugated π-conjugated structure of conductive polymers can also carry out electron transfer and commonly used polyaniline. Polyaniline, polypyrrole, and PEDOT: PSS are commonly used. Due to the good biocompatibility of polyaniline, polyaniline-based CHs are often used in the field of biomedicine. For example, in situ polymerization of aniline in an aqueous solution of poly(2-acrylamide-2-methyl-1-propane sulfonic acid), followed by poly(ethylene glycol) cross-linked redox CHs can be used for electro-oxidation of glucose [57].

Apart from the introduction of a single type of conductive material mentioned above, a variety of conductive materials can also be mixed and integrated into a system to achieve the desired electrical conductivity and mechanical properties.

#### 2.2.2. I-CHs

The polymer networks of the hydrogels are rich in a large amount of freely mobile water, which provides an excellent environment for the migration of ions [58]. There are three main classes of materials that can generate free ions in water to achieve electrical conductivity: metal salts (e.g., NaCl, KCl, LiCl, FeCl_3_, AlCl_3_); acids (e.g., HCl, H_2_SO_4_); ionic liquids (e.g., 1-ethyl-3-methylimidazolium chloride). Unlike E-CHs, which usually have darker colors, I-CHs mostly have the advantage of high transparency due to their ionic properties. In addition, the functional design of mobile ions in I-CHs is well suited as electrolytes for solid-state batteries.

Metal salts are often used to prepare I-CHs. For example, Chen et al. [59] prepared CHs flexible electrodes based on NaCl/LiCl and PAM and applied them to a transparent actuator. When a voltage is applied, such an actuator converts electrical energy into mechanical deformation. The ions in the acid can also achieve electrical transfer. For example, the introduction of H_2_SO_4_ into PVA and PVP hydrogels, respectively, can lead to high-performance solid electrolytes and self-healing supercapacitors [60,61]. Compared with metal salts and acids, ionic liquids are used in fewer examples, but the performance is equally excellent. Liu et al. [39] prepared self-healing CHs synthesized by UV light self-initiation method based on 1-ethyl-3-methylimidazolium chloride. It provides an efficient platform for CHs electrolytes to obtain tunable supercapacitor performance for energy storage devices.

#### 2.2.3. E-CHs and I-CHs

In addition to introducing a single species of electronic/ionic conductive materials, they can be simultaneously integrated into the same hydrogels system to combine their advantages [47]. For example, conductive polymers (e.g., PEDOT: PSS, polyaniline) and acids (e.g., HCl, H_2_SO_4_) can be used to prepare flexible electrodes and supercapacitors [40,62,63], metal salts (e.g., NaCl, MgCl_2_, CaCl_2_) and carbon-based materials (e.g., CNTs, rGO) can be used to prepare pressure sensors and strain sensors [41,64,65]. There are still many similar mixes and matches between electronic and ionic conductive materials, and the resulting CHs can be surprisingly good in some aspects.

## 3. General Methods for Synthesizing CHs

The synthesis of CHs directly affects their physical and chemical properties and mechanical performance, and further affects their applications. To synthesize CHs that meet application needs, different organics, inorganics, and/or conjunct polymers are combined to maximize the value of these feedstocks. According to the different synthesis mechanisms, the general techniques for synthesizing CHs can be divided into polymerization techniques and cross-linking techniques [1].

### 3.1. Polymerization Techniques

Synthetic polymers generally have more stable chemical structures than natural materials, as do hydrogels. As with other cross-linked polymers, for the synthesis of hydrogels, any method that can be used to produce cross-linked polymers is equally applicable. The polymerization methods have a significant impact on the properties of the prepared hydrogels and are a key part of the preparation of CHs [66]. In general, hydrogels can be prepared by two polymerization methods. The preparation of hydrogels could be achieved either in a uni-step procedure via polymerization of the polyfunctional monomers with concurrent crosslinking of them or in a multi-step procedure that includes the assembly of reactive polymers that can be crosslinked by themselves or via reaction with suitable crosslinkers [62,67]. The following subsection discusses the different polymerization techniques used to prepare hydrogels.

#### 3.1.1. Chain Growth Polymerization Method

The chain growth polymerization method is essentially a free radical polymerization method and is often used to prepare chemical cross-linking hydrogels. During the chain growth polymerization, carbon-carbon double bonds involving hydrophilic monomers are often shared in free radical polymerization [58]. Several methods for preparing hydrogels by free radical polymerization include bulk polymerization, solution polymerization, suspension polymerization, emulsion polymerization, and graft polymerization.

The bulk polymerization method is relatively simple in principle and operation, so it is widely used in the preparation of hydrogels. This method relies on the polymerization of fluid monomers and an initiator, where the initiator needs to be soluble in the monomer with a small amount of cross-linking agent. In the process, it is generally necessary to rely on external conditions to carry out the aerodynamic polymerization process, including chemical catalysts and ultraviolet radiation [63]. Although bulk polymerization has the advantages of simple principle and operation, the effect is not ideal, and it is difficult to meet the application requirements. Compared with bulk polymerization, suspension polymerization and emulsion polymerization provide better control of the heat generated during polymerization and are easier to design [64]. Other polymerization methods, such as solution polymerization and graft polymerization, are also superior to bulk polymerization in terms of effect. Therefore, they are more commonly used in hydrogels synthesis.

The solution polymerization method utilizes redox reactions or ultraviolet radiation to promote the pneumatic polymerization process of monomers (neutral or ions), solvents (e.g., water, benzene, ethanol, or water-ethanol mixtures), and multifunctional crosslinkers. After the polymerization process is over, the hydrogel polymers are separated, and the remaining unreacted components (cross-linking agent, monomer, initiator, etc.) are washed away with distilled water [65]. The solution polymerization method has many advantages, including ease of synthesis, optimized thermal control methods, safety, and a cost-effective polymerization process [68]. Firstly, this method can be carried out at room temperature, and the polymerization frequency is high. Furthermore, due to the lower viscosity of the solution, the uniform mixing of the reaction components in solution polymerization is easier compared to bulk polymerization, resulting in enhanced heat dissipation [69]. Hence, solution polymerization is often used to prepare hydrogel polymers with excellent properties.

The suspension polymerization method is to continuously stir insoluble monomers and low hydrophilic-lipophilic equilibrium initiators in a solution to generate droplet particles with a diameter of 0.1–5 mm, and then use filtration technology to separate them from various reaction components [70]. As the same as the solution polymerization method, the heat transfer rate of this method is enhanced by the presence of water. In addition, the suspension polymerization process also utilizes colloids (e.g., PVA, methyl cellulose (MC)) to prevent adhesion between droplets.

Polymers prepared by emulsion polymerization are smaller compared to polymers prepared by suspension polymerization. The reactive components of this process include water-soluble small monomers (fully hydrophobic or partially water-soluble monomers), water-soluble initiators, cross-linking agents, and surfactants [71]. The polymer droplets produced by this method are also smaller than those produced by suspension polymerization. Like suspension polymerization, emulsion polymerization is also easy to adjust and has better heat transfer capabilities than bulk polymerization.

Generally, hydrogels synthesized by bulk polymerization have poor mechanical properties, but graft polymerization can make up for this shortcoming. The graft polymerization method is mainly to graft on a solid support frame to improve the mechanical properties of the hydrogel. Free radicals are generally formed in the region of the support, where monomers polymerize and create stronger covalent bonds with the support shell. For example, vinyl monomers are often grafted onto polysaccharides to increase their mechanical strength [67].

#### 3.1.2. Step-Growth Polymerization Method

The step-growth polymerization method utilizes monomers with certain functional groups to initiate a single-step polymerization reaction through covalent bonds to synthesize hydrogels. The mechanical properties (integrity, ductility, tensile strength, and shear strain) of the hydrogels prepared by this method are superior to those synthesized by the chain-growth polymerization method, which is due to the synergy of the network and the qualitative.

In general, the chain-growth polymerization method and step-growth polymerization method have their advantages and disadvantages and can be appropriately selected according to actual needs and conditions. An accurate understanding of hydrogel cross-linking and networking in principle will facilitate the synthesis of hydrogels with expected properties.

### 3.2. Cross-Linking Techniques

Hydrogels are formed by polymeric cross-linking since cross-linking can slow down the decomposition of the hydrophilic elements of the hydrogel polymers in the aqueous medium. The larger the amount of cross-linking, the higher the elasticity and viscoelasticity of the hydrogel polymers, so hydrogels can exhibit the dual properties of solid and viscoelastic [72,73]. The cross-linking ratio has a close effect on the properties of the synthesized hydrogels [74]. Furthermore, hydrogels can be obtained by different cross-linking methods, generally referred to as physical cross-linking (physical reaction to synthesize polymer network) and chemical cross-linking (chemical reaction to covalently bond to form polymer networks) [75]. Their advantages and disadvantages are presented in Table 2. The type of cross-linking of the hydrogel networks is a key factor in analyzing the structure of the hydrogels and the basis for them to exhibit different properties. Therefore, identifying a suitable cross-linking method and adjusting the degree of cross-linking facilitates the synthesis of hydrogels with application-specific properties for a wide range of biomedical applications.

#### 3.2.1. Physical Cross-Linking Method

Physical cross-linking hydrogels are synthesized through a variety of physical interactions, including ionic/electrostatic reactions, hydrophilic interactions, hydrophobic interactions, hydrogen bonding, protein interactions, metal coordination, and crystallization [76,82,83,84,85]. These physical interactions between polymer chains do not involve the formation of covalent chains and are therefore reversible [86]. For example, the compounds synthesized by the reaction of polymethacrylates and polyacrylates with PEG with carboxylic acid groups through hydrogen bonding are pH-responsive, so they can be made into pH-responsive CHs. Because physical cross-linking is reversible, its stability under physiological conditions will be significantly reduced or even zero, which greatly limits the application prospects of physical cross-linking hydrogels in the field of biomedicine. Hence, chemical cross-linking hydrogels are a viable alternative for applications in the field of biomedicine.

#### 3.2.2. Chemical Cross-Linking Method

Bulleted lists look like this: Unlike physical cross-linking, chemical cross-slinking is usually an irreversible reaction process that forms covalent bonds. With the help of covalent building, chemical cross-linking hydrogels have higher stability and mechanical properties than physical cross-linking hydrogels in a physiological state. Although chemical cross-linking is irreversible, the biodegradability of chemical cross-linking hydrogels is tunable [86]. Chemical cross-linking methods mainly include photopolymerization, thermal polymerization, enzyme cross-linking, etc. [87,88,89]. For example, acrylic derivatives with glycidyl methacrylate (GMA) and polyethylene glycol diacrylate (PEGDA) as unsaturated groups were used to synthesize hydrogels by photopolymerization and cross-linking [90]. And PEG/hyaluronic acid hydrogels are generated by transglutaminase for biomedical cell and tissue regeneration [91].

The “Click chemistry” method is a new “chemical cross-linking” method, which solves some problems of traditional chemical cross-linking to a certain extent. The basic idea of “Click chemistry” is to use carbon-heteroatom bonding reactions to rapidly achieve molecular diversity, generally by the interaction of azide and alkyne to form covalent bonds. The reaction process is not affected by pH and can be carried out in the water at room temperature, even in living cells. The proposal of “click chemistry” complies with the requirements of chemical synthesis on molecular diversity and promotes the development of chemical synthesis technology [81,92].

Although chemical cross-linking has many advantages over physical cross-linking, there are also some problems. Some toxic cross-linking agents or reagents are used in chemical cross-linking, which can cause environmental problems and bio-identity safety issues. Both physical cross-linking and chemical cross-linking have their own advantages, and it is the best choice to choose the appropriate method to prepare hydrogels according to actual needs [80,93].

## 4. Development and Applications of CHs in the Field of Biomedicine

The 3D porous structure, hydrophilicity, tunable physical and chemical properties, and excellent biocompatibility of CHs are very similar to the extracellular matrix of biological tissues. In addition, the excellent electrical conductivity and electrochemical redox properties of CHs can be exploited to monitor bioelectrical signals or provide electrical stimulation for the regulation of biological activities of cells/tissues [94]. These advantageous properties make CHs widely used in the field of biomedicine, including regenerative medicine [95,96,97], artificial organs [16,98], biosensors [99,100,101], drug delivery systems [102,103,104] as well as surgical treatment, wound dressings and other applications [105,106,107,108] (Table 3).

### 4.1. Regenerative Medicine

Regenerative medicine refers to the use of theoretical methods of biology and engineering to repair or regenerate damaged or lost tissues and organs so that they have the structure and function of normal tissues and organs [126]. By studying the normal tissue characteristics and functions of the human body, the mechanism of wound repair and regeneration, and the mechanism of stem cell differentiation, researchers are seeking effective biological treatment methods to promote the body’s self-repair and regeneration, or to construct new tissues and organs to maintain, repair, regenerate or improve the function of damaged tissues and organs. Regenerative medicine has a broad connotation, including tissue engineering technology and biological active platforms, biological scaffolds, and tissue-like materials used in the process.

The main goal of tissue engineering is to repair damaged tissues and replace them with new biological tissues [10]. In the repair process, biocompatible bioactive platforms and scaffolds are often required to assist [127]. CHs have 3D porous structures that can attach bioactive components (e.g., biomolecules, proteins, growth factors) and are biocompatible. Therefore, the bioactive platform based on CHs can be used as a carrier to transport the biological components required for tissue regeneration to control and promote tissue regeneration and development. CHs-based scaffolds can bridge tissue defects by stimulating new tissue growth and neovascularization while exhibiting a high degree of fusion and biodegradation, they can disappear during or after healing is complete [127].

**Figure 2:**
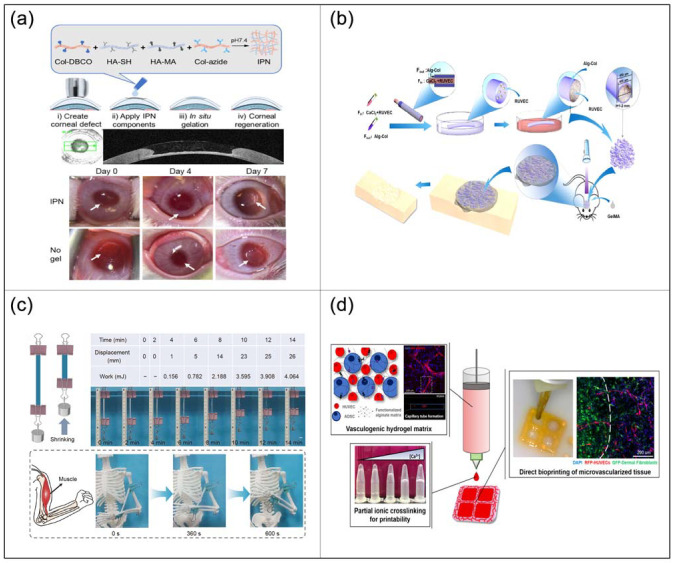
Applications of CHs in regenerative medicine and artificial organs. (**a**) Ex vivo and in vivo studies of the application of the IPN as a corneal defect filler. (**b**) Schematic illustration of the fabrication and application of BMVs loaded with RUVECs. (**c**) Application experiment of artificial muscle. (**d**) Printing artificial blood vessels using hydrogel bioinks.

The eyes are one of the most important organs of human beings and the bridge between human beings and the real world, so blindness is one of the most tormenting diseases. Corneal damage is the leading cause of blindness, and timely treatment of corneal damage can help prevent blindness. Novel hydrogel developed by Chen et al. [128] can fill corneal defects and assist corneal regeneration (Figure 2a). This hydrogel is a simultaneous interpenetrating polymer network (IPN) composed of collagen cross-linked via strain-promoted azide–alkyne cycloaddition reaction and hyaluronic acid cross-linked via thiol-ene Michael clicks reaction. IPN combines the advantages of collagen and hyaluronic acid gels to avoid epithelial hyperplasia and promote corneal tissue regeneration. To restore intact and clear corneas, bio-integrated materials are also required to repair and replace corneal tissue in situ. Yazdanpanah et al. [129] developed a light-curable cornea matrix (LC-COMatrix) bio-integrated material. This is a functionalized hydrogel based on a porcine corneal extracellular matrix and is biodegradable. It can also be cross-linked in situ under visible light with significantly enhanced biomechanical strength, stability, and adhesion. LC-COMatrix can adhere to the human cornea, effectively closing corneal perforations and tissue loss and replacing the partially missing tissue. This bio-integrated material is representative of natural corneal stroma and has potential application value in ocular tissue engineering.

Bone tissue engineering is one of the most difficult tissue engineering techniques, and insufficient vascularization is one of the key factors leading to unsatisfactory bone repair results. Currently, building vascular networks and maintaining their activity remains a challenge. Wang et al. [130] used microfluidic technology to prepare biomimetic microvessels (BMVs) loaded with rat umbilical vein endothelial cells (RUVECs) in one step (Figure 2b). This BMV supports perfusion and extravasation of internal substances, and RUVECs can adhere and proliferate on the BMV inner wall. At the same time, BMV can also promote bone regeneration. This study not only provides a new type of bone repair material but also provides a new idea for vascularization strategies in tissue engineering.

Different from common hard bone tissue, the repair of cartilage tissue presents special technical difficulties. Ishikawa et al. [131] designed a one-step in situ gelation system to construct the precursors of IPN gels on demand. The design involves two gel processes: self-assembly of the RADA16 polypeptide and covalent bond formation between chitosan (CH) and N-hydroxysuccinimide ester-terminated poly(ethylene glycol) (NHS-PEG-NHS). The chondrocytes were cultured with CH/PEG/RADA16 and the results showed that the construction of the IPN structure promoted the properties of embedded chondrocytes, which was beneficial to the formation of articular cartilage without causing inflammation. This demonstrates the feasibility of designing IPN hydrogels as bioscaffolds for cartilage tissue engineering.

### 4.2. Artificial Organs

Artificial organs are a technically difficult but necessary research topic in the field of biomedicine. Those whose organs are damaged or even fail due to diseases or accidents need artificial organs to maintain life.

The muscle is the main organ that performs body movement and is also an important organ of body energy metabolism. There are more than 600 muscles in the human body, and their weight accounts for about 40% of the body weight. The application of artificial muscles can help those suffering from muscle diseases to restore normal exercise capacity. Hua et al. [132] developed an ultra-strong programmable artificial muscle based on a cryogenic shape memory hydrogel. The material is composed of polyacrylic acid hydrogel and calcium acetate complex. It is soft and editable at room temperature and hardens when heated to 70 °C to fix the shape. When placed at low temperature again, the material softens and returns to its original shape. They developed the material into a biological muscle model, where the bone can lift the arm when contracted at low temperatures (Figure 2c). Its functional capacity is as high as 45.2 kJ m^−3^, far exceeding that of general animal muscles (~8 kJ m^−3^). This study shows that the material has certain application prospects in artificial muscles.

The kidney organ is a high-risk disease area, and the directed differentiation of human pluripotent stem cells brings the prospect of an artificial kidney organ. Currently, this application is limited by organ diversity, nephron immaturity, and limited scale. To address these issues, Lawlor et al. [133] developed an extrusion-based 3D bioprinting to construct artificial kidney organs. This technique enables precise manipulation of biophysical properties, including organ size, cell number, and conformation. As a result, the extrusion-based 3D bioprinting technique for artificial kidney organ production has improved in yield, quality, scale, and structure, facilitating the in vivo and in vitro application of stem cell-derived human kidney tissue. Besides kidneys, intestinal organoids are also core functional human organs. Chrisnandy et al. [134] developed CHs with hybrid networks structure based on PEG and cytosine, which can promote organoid morphogenesis through dynamic rearrangement mediated by reversible hydrogen bonds, especially for the artificial culture of intestinal organoids.

In addition to kidney and intestinal organoids, blood vessels are also unique organs, and the development of artificial blood vessels is a key challenge in the field of biomedicine. Although protein-based hydrogel inks have been successfully used to bioprint microvascular systems, their composition is unclear and prone to batch variation. Barrs et al. [135] developed an alginate hydrogel bioink. The ink has well-defined mechanical, rheological, and biochemical properties for direct bioprinting of microvascular tissues (Figure 2d). They used this bioink to create artificial blood vessel tissue and observed new blood vessel tissue regeneration. This study provides a tunable and translatable hydrogel bioink with defined composition for the fabrication of artificial blood vessels. But current bioinks and printing methods have several shortcomings that limit the ability to print elastic and highly vascularized tissues. To address these issues, Lee et al. [136] used recombinant human orthoelastin as a highly biocompatible and elastic bioink. They used this ink to print a vascularized heart construct. The cardiomyocytes of the printed artificial vascularized heart have endothelial barrier function and can spontaneously beat, realizing the function of the heart. In addition, the ink has virtually no inflammatory response and is biodegradable. This elastic bioink has application potential in printing functional artificial vascular tissue and artificial heart tissue, which can be used for artificial heart replacement.

### 4.3. Biosensors

Biosensors are of great significance in biological research and the diagnosis and treatment of human diseases. Based on the biosensor monitoring data, we can update the treatment plan in real-time to achieve the desired goal. Biosensors can be divided into wearable and implantable biosensors according to their different use modes, both of which can play an important role in the field of biomedicine.

#### 4.3.1. Wearable Biosensors

**Figure 3:**
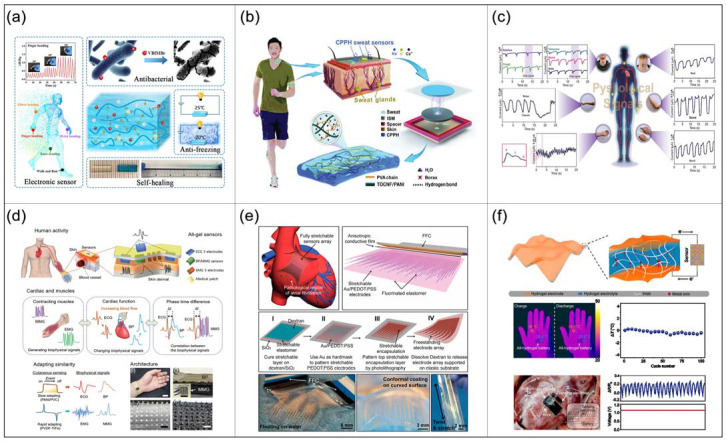
Applications of CHs in biosensors. (**a**) Principle and application of a novel ionic liquid segmental polyelectrolyte CHs. (**b**) Cellulose-based conductive hydrogel is used for self-powered sweat sensing. (**c**) Physiological data health monitoring of different parts by electronic skin. (**d**) Schematic diagram of the artificial skin sensor system and heart-related biophysical signals. (**e**) Fabrication and assembly of elastomeric arrays for cardiac health monitoring. (**f**) Schematic illustration of the structure and working mechanism of the all-hydrogel battery; application of the all-hydrogel batteries.

Wearable biosensors are relatively common, and their biggest feature is portability, so they are widely used in the field of biomedicine and daily life. For example, Zhou et al. [137] prepared a novel ionic liquid segmented polyelectrolyte CHs composed of acrylic acid (AAc), 1-vinyl-3-butylimidazolium bromide (VBIMBr), and Al^3+^ (Figure 3a). The maximum tensile strength is 0.16 MPa, the maximum fracture strain is 604%, and the electrical conductivity is 12.5 S/m. This kind of CHs can stably and sensitively monitor human motion status even in extreme environments (−20 °C), which has great application potential in healthcare monitoring. For motion monitoring, Lin et al. [138] combined red fluorescent hydrogels, polydimethylsiloxane, and CNTs to make a dual-channel flexible biosensor for human motion monitoring.

“Feeling the pulse” of traditional Chinese medicine (TCM) is magical and effective. Wang et al. [139] proposed a wearable multi-channel pulse monitoring biosensor based on TCM pulse theory. The device can simultaneously detect the pulse state of three pulse positions (inch, close, and chi), and provides a 3D pulse map, which vividly reveals the shape of the pulse length and width, making up for the shortcomings of traditional single-point pulse sensors.

In medical and daily health monitoring, visualization of sweat composition is critical for physiological assessment, but the material’s insufficient healing ability and unreliable power supply methods limit its application. Qin et al. [140] fabricated a self-powered sweat sensor using the developed cellulose-based CHs to address these issues (Figure 3b). Among them, the CHs electrodes consist of cellulose nanocomposites based on PVA and PANI. This material provides a sweat sensor with over 95% stretch and electrical self-healing efficiency within 10 s, a 1530% stretch ratio, and 0.6 S m^−1^ conductivity. The sweat sensor quantitatively analyzes Na^+^, K^+^, and Ca^2+^ content in sweat in real-time through the triboelectric effect, and wirelessly transmits the results to the user interface. This sweat sensor exhibits high flexibility, stability, sensitivity, and selectivity, providing new opportunities for self-powered biomedical health monitoring.

Electronic skins are the most ideal form of wearable biosensors but require self-healing capabilities to maintain robustness and reliability. Lin et al. [141] developed a CHs electronic skin with self-healing ability by mixing carbon-based materials with cellulose nanofibers-PVA-hydrogels. This electronic skin can be worn on different body parts (e.g., throat, wrist, back of neck) to achieve different physiological data monitoring purposes (Figure 3c). At the same time, the electronic skin can also be connected with traditional electronic devices through an interface, and visualization can be realized via Bluetooth. Similarly, Su et al. [142] developed cutaneous CHs based on the hydrophobic association between poly(acrylic acid) (HAPAA) and PANI. This hydrogel exhibits good mechanical properties that perfectly match human soft tissues, with Young’s modulus of about 128 kPa, a toughness of about 15.66 MJ m^−3^, fracture energy of about 13.99 kJ m^−2^, a tensile strength of about 1.35 MPa, compressive strength of about 23.1 MPa and high ductility of about 2150%. In addition, it is freeze/heat resistant, self-healing, and has multiple sensing modalities (stretch, pressure, temperature). Combining the above advantages, this kind of CHs has broad application prospects in the field of biomedicine. Wearable sensors for monitoring heart health have always been a technical challenge. Chun et al. [143] developed a wearable artificial skin biosensor based on CHs. This biosensor consists of four-element sensors that simultaneously collect blood pressure, electrocardiogram (ECG), electromyogram (EMG), and mechano-myogram signals related to cardiac and muscle health (Figure 3d). Monitoring the relationship between muscle and heart is critical for screening and predicting heart health, so this multimodal skin-like sensor has outstanding applicability in monitoring physiological conditions and diagnosing heart health problems.

#### 4.3.2. Implantable Biosensors

Unlike wearable biosensors, implantable biosensors need to be implanted into the human body to function. It also means that implantable biosensors can perform data monitoring tasks more directly and in detail, often with higher accuracy.

Heart disease has been the “No.1 killer” in recent years, accounting for 16% of all deaths. The application of implantable sensors can effectively provide information on heart health status and give early warning of heart diseases. Liu et al. [144] developed a tissue-like, high-density, fully elastic implantable microelectrode array based on I-CHs (Figure 3e). This microelectrode array can record complex electrophysiological signals in vivo at cell-level resolution in real-time and stably, and assist in electrophysiological mapping, which is helpful to clarify atrial fibrillation (AF) mechanism at the cell level and develop targeted AF therapy. Similarly, Gu et al. [145] also developed an implantable flexible sensor array that can monitor the conduction of electrical signals within/between cells of cardiomyocytes to monitor and prevent heart disease. The tiny “pop-up” sensors enter cells without damaging them and directly measure the conduction and velocity of electrical signals within individual heart cells, as well as obtain high-resolution pictures of the heart. This achievement can replace the traditional patch-clamp technology to a certain extent in the future for the monitoring of intracellular electrophysiological signals, and provide technical support for the study of the electrical signal activity inside neurons.

#### 4.3.3. Energy Supply for Biosensors

Energy devices are crucial for the stable operation of biosensors. Batteries have the advantages of high energy density and recyclability and are the first choice for energy devices. Biosensors require batteries with matching mechanical properties (e.g., Young’s modulus) to biological tissues. This not only facilitates the normal operation of the biosensor but also reduces irritation and damage to the tissue, thereby avoiding immune responses and health hazards. Hence, tissue-like soft batteries are of great significance for the development of biosensors. Ye et al. [146] developed an integrated battery configuration with excellent electrical conductivity and high interfacial charge transfer efficiency based on self-developed CHs and formed a general approach to achieve ultrasoft batteries with high electrochemical performance (Figure 3f). At a current density of 0.5 A g^−1^, the specific capacity of the lithium-ion battery based on self-developed CHs is 82 mAh g^−1^, and the specific capacity of the zinc-ion battery is 370 mAh g^−1^. Meanwhile, this cell exhibits a tissue-like Young’s modulus of 80 kPa, which can be perfectly matched to organs such as skin and heart, and its biocompatibility has been demonstrated in practical applications. This research provides a practical way to address the mechanical mismatch between biosensors and biological tissues.

Besides external battery power supply, self-powered biosensors are also a new idea. Xia et al. [147] developed a self-powered ionized skin biosensor based on gradient polyelectrolyte membranes (GPMs), which can directly and accurately sense a variety of stimuli. The GPMs prepared by the hydrogel-assisted reaction-diffusion method exhibit a gradient distribution of charged groups in the polymer network, which enables the generation of thickness-dependent and thermally responsive self-induced potentials for self-powering. Furthermore, the coupling of mechanoelectrical and thermoelectric effects inherent in GPMs provides a general strategy for developing ion-based self-powered sensing systems.

### 4.4. Drug Delivery Systems

In the field of biomedicine, to achieve the desired therapeutic effect and avoid side effects, safe and reliable drug delivery systems are often required. The functional design of CHs provides favorable conditions for drug encapsulation, protection, and delivery [148]. In addition, they are biocompatible and biodegradable to avoid immune rejection.

The controllable drug delivery systems with high efficiency, precise release position, and release time are the future trend of drug delivery systems development. Mauri et al. [149] used the dynamic light scattering (DLS) technique to prepare biodegradable poly(ethylene glycol)-block-poly(lactic acid) (PEG-b-PLA) nanoparticles (NPs) by nanoprecipitation with controlled size and different charges. Then, NPs were loaded into CHs for the central nervous system. They investigated the ability of the composite hydrogels-NPs system to control the release of small sterically hindered drugs. Through experiments, research, and later model analysis, they were able to develop different drug delivery devices to meet different medical needs.

**Figure 4:**
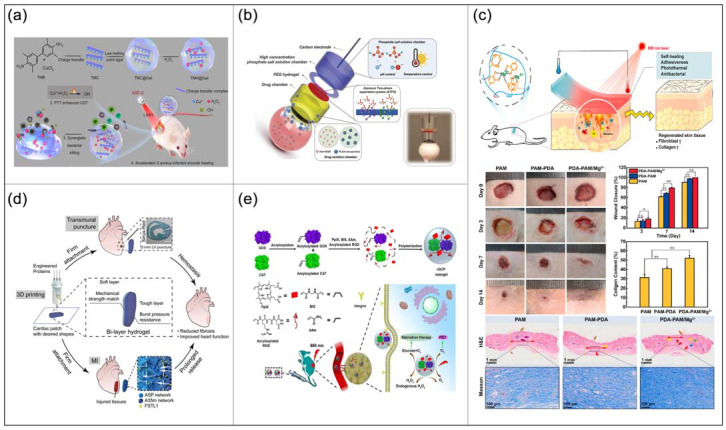
Applications of CHs in drug delivery systems and other biomedical fields. (**a**) Schematic illustration of the preparation of TMH@Gel Complex and the mechanism of TMH@Gel for accelerated wounds healing. (**b**) Schematic of a high-intensity iontophoresis device for intraocular delivery of macromolecules and nanoparticles. (**c**) Schematic illustration for the mechanism and application of PDA-PAM/Mg^2+^ hydrogel in wound healing in a full-thickness defect model; Wound healing effects in vivo of the hydrogels. (**d**) Schematic diagram of the working principle of the minimally invasive cardiac patch. (**e**) The synthetic route of rGCP nanogels and the application in amplified synergistic cancer therapy by combining starvation and photodynamic therapy.

Bacterial infection is a worldwide problem that seriously threatens human health. Building a multifunctional system that can synergize antimicrobial therapy is a challenging task. Niu et al. [150] developed a drug delivery CHs TMH@Gel with near-infrared-II (NIR-II) absorption function and high photothermal conversion (Figure 4a). Under NIR-II light irradiation, TMH@Gel exhibited excellent antibacterial effects against both Gram-negative and Gram-positive bacteria through synergistic photothermochemical kinetic treatment. When TMH@Gel is used in the drug delivery system, the drug and antibacterial effects can significantly accelerate the healing of bacterially infected wounds. The achievement also provides a feasible idea for the development of antibacterial drug delivery systems.

Iontophoresis is an electrical current-based non-invasive drug delivery technology that is especially suitable for intraocular drug delivery. However, current iontophoresis techniques use low-intensity currents to avoid damage to ocular tissues, which greatly limits the delivery efficiency of macromolecular and nanoparticle drugs. To address this limitation, Zhao et al. [151] developed an iontophoresis device based on phosphate solutions and PEG hydrogels (Figure 4b). The device can safely apply high-intensity currents (up to 87 mA cm^−2^, more than 10 times higher than current ocular iontophoresis devices) to the eyes with virtually no damage. In addition, the device significantly improves the delivery efficiency of macromolecules and nanoparticles (up to 300 times). At the same time, the device can be easily operated by ordinary nursing staff, which is very beneficial to popular eyes treatment.

The treatment of cancer has always been a focus of medical research, and various macro/micro biomaterials have been developed for drug delivery in tumor therapy. However, uncertainties in drug loading ratio, release order, and spatiotemporal distribution hinders their synergistic therapeutic effects and clinical applications. Based on this, Li et al. [152] developed a tumor microenvironment-adaptive composite consisting of thermosensitive hydrogels and reactive oxygen species (ROS)-responsive nanogels. It can be used for precision drug delivery to enhance molecularly targeted therapy and amplify immune activation. The application of this material can effectively inhibit tumor growth and liver metastasis, which is of great significance for improving the prognosis of patients with advanced cancer.

### 4.5. Other Biomedical Applications

Besides regenerative medicine, artificial organs, biosensors, and drug delivery systems, CHs have many other applications in the field of biomedicine, such as wound dressings and cancer therapy.

#### 4.5.1. Wound Dressings

Bacterial infection is an important factor in delaying wound healing, and antibiotics can lead to resistance to pathogenic bacteria. Therefore, there is an urgent need for non-antibiotic antibacterial biomaterials with enhanced wound healing properties in the current biomedical field. Di et al. [153] designed a transparent wound dressing using bacterial cellulose (BC) and poly(2-hydroxyethyl methacrylate) (PHEMA) CHs. This dressing is antibacterial and promotes aseptic wound healing. Furthermore, Guo et al. [154] developed a composite antibacterial PDA-PAM/Mg^2+^ hydrogel based on PDA and PAM. It shows excellent self-healing and tissue adhesion properties and has photothermal antibacterial functions that can promote wound healing (Figure 4c). Its self-healing property extends the service life of the dressing, effectively ensuring wound closure and preventing infection. The photothermal effect can stimulate blood circulation, inhibit bacteria and reduce inflammation. This antibacterial hydrogel has broad potential in clinical adjuvant therapy.

In addition to external dressings, the development of internal minimally invasive cardiac patches, whether as hemostatic dressings or for the treatment of heart disease, has great clinical significance. Jiang et al. [155] developed a CHs patch for heart failure treatment using genetically engineered multidomain proteins (Figure 4d). It is non-toxic, matches the mechanical properties of cardiac tissue, and is biodegradable without causing inflammation. This minimally invasive patch can be used as a cardiac hemostatic dressing or to treat heart diseases.

#### 4.5.2. Cancer Treatment

Cancer has always been recognized as a medical problem in the world and a chronic disease in the field of biomedicine. Tumors often require a large number of nutrients to maintain the rapid growth of cancer cells, so starvation therapy based on the principle of cutting off the supply of glucose is an effective treatment. However, starvation therapy is often inhibited by the intrinsic tumor hypoxic microenvironment. To overcome this limitation, Fan et al. [156] developed a composite enzyme nanogel rGCP with self-oxygenation ability that can synergize starvation and photodynamic therapy (Figure 4e). The rGCP nanogel was fabricated by copolymerizing two monomers, porphyrin, and cancer cells-targeted, Arg-Gly-Asp (RGD), onto the glucose oxidase (GOX) and catalase (CAT) surfaces. rGCP provides a new method for constructing nanomedicine combination therapy with good tumor targeting and therapeutic effect.

In the treatment of cancer, patient-derived tumor organoids (PDOs) are a promising preclinical model that recapitulates the histology, gene expression, and drug response of donor patient tumors. Currently, the cultivation of PDOs relies on basement-membrane extract (BME), which suffers from batch-to-batch variability and poor control of mechanical properties. To address these issues, Prince et al. [157] developed a nanofibrous hydrogel (EKGel) for the culture of breast cancer PDOs. PDOs grown in EKGel have similar histopathological features, gene expression, and drug responses to their parental tumors. In addition, EKGel reduces batch-to-batch variability, provides controlled mechanical properties, and inhibits cell contamination. Based on the above conditions, EKGel can better replace BME for the culture of breast cancer PDOs.

Radiotherapy radiation in cancer treatment is as harmful to human health as cancer itself, so it is very necessary to reduce the harm of radiotherapy radiation. Guan et al. [158] fabricated hydrophobic alkene CHs with various desirable characteristics based on different supramolecular assemblies. Such CHs exhibit excellent electrical conductivity, super stretchability (>2000%), and self-adhesion, which can be used to separate tumors from surrounding organs during brachytherapy to reduce damage. The team also developed an application to monitor the safe range of different radiation risks, showing its great potential in soft smart sensors.

## 5. Summary and Prospects

CHs feature excellent electrical conductivity, flexibility, transparency, biocompatibility, and other characteristics, making them incomparably advantageous in the field of biomedicine. In order to provide a reference for those who are interested in CHs, this review summarizes the properties of CHs, preparation materials, and synthesization methods, and introduces their various applications in the field of biomedicine. In recent years have witnessed exponentially increased research and applications of CHs. With continuous efforts, some exciting results have been achieved, but there is still a lot of room for development in the related research and applications of CHs. Based on the current development of CHs and their applications in the field of biomedicine, this section proposes several issues that need to be focused on and gives an outlook on the possible future directions of the development of CHs.

### 5.1. 3D Bioprinting of Microtissue Structures

At present, the application of CHs relies on 3D bioprinting technology to complete the configuration in many cases. Many biomedical applications involve microstructures, but the current 3D bioprinting technology is difficult to fabricate microstructures. We can learn from micromachining technology and soft lithography technology to develop 3D bioprinting technology that can print biological microtissue structures to meet the application needs in the field of biomedicine.

### 5.2. Practical Biomedical Application Progress of CHs

No matter how many advantages they have, current CHs only remain in theory and on paper, and only the real clinical applications can give full play to their advantages. Currently, the adaptation of CHs to ongoing physical, functional, and pathological changes remains a challenge. Therefore, there is still a need to develop novel CHs materials with smart properties to facilitate clinical applications in the field of biomedicine.

CHs have been practically applied in many scenarios in the field of biomedicine, but there is still a certain gap from the most efficient systematic applications. The properties of CHs make them widely used in regenerative medicine, artificial organs, drug delivery systems, etc., but they are all limited to a single technical application. If the applications of CHs in various aspects of the biomedical field are integrated into a complete set of systematic technologies, the effect will leap, and their advantages will be more prominent.

### 5.3. Programmable CHs

The design and preparation of CHs with programmable functions is another interesting topic for future research. For different initial inputs, programmable CHs can have programming content corresponding to different outputs and can be modified in real-time. Programmable CHs can also be erasable like traditional chips, changing their internal programs on demand. Hence, programmable CHs can realize complex actions such as self-bending, twisting, and folding postures to complete more difficult biomedical applications such as surgery and drug delivery, and have a significant effect on improving work efficiency and accuracy.

### 5.4. Cross-Applications of CHs in Frontier Fields

Supercomputing, big data, and artificial intelligence (AI) are all frontier fields in today’s world, and the cross-applications of CHs in the biomedical field and these frontier technology fields will be a hot topic. Combine biomedical applications (e.g., biosensors, drug delivery systems) with supercomputing, big data, and AI to realize digital and intelligent biological health monitoring, drug delivery, and other functions. In this way, both intuitive observation and accurate control can be achieved, giving full play to the advantages of various fields.

The research of CHs in the field of biomedicine has achieved a lot of results, and the performance has reached a relatively high level, but there are still some key theoretical and technical problems to be solved. For example, the further improvement of biodegradability, how to achieve complete biocompatibility, how to improve their working stability and compatibility with electronic components, etc., are the focus of biomedical applications based on CHs in the future.

The biomedical research and applications of CHs involve materials, mechanical, biology, medicine, and other disciplines. Progress in each discipline direction will be a catalyst for the development of CHs. It is foreseeable that in the future, CHs will shine in the field of biomedicine and become as indispensable as scientific research talents.

## Figures and Tables

**Table 2:** Advantages and disadvantages of physical cross-linking and chemical cross-linking technology.

Types of Cross-Linking	Advantages	Disadvantages	Refs
Physical cross-linking (Non-permanent)	(1) The synthesis principle is a physical action, which is relatively simple; (2) No need to use toxic cross-linking agents; (3) The method can be improved according to the actual situation to improve the properties of the hydrogel.	(1) The resulting hydrogels generally have poor mechanical properties; (2) There may be uneven swelling distribution; (3) The number of components that can be cross-linked is limited	[1,76,77,78]
Chemical cross-linking (Permanent)	(1) The resulting hydrogels are generally structurally stable and have good mechanical properties; (2) Unlimited number of components that can be cross-linked; (3) It has substrate specificity, keeps effective reactions continuous, and avoids ineffective reactions; (4) “Click chemistry” has high efficiency, stability, high specificity, and high control, and it meets molecular diversity requirements.	(1) Toxic crosslinking agents are required in the chemical cross-linking process; (2) There may be uneven swelling distribution; (3) Chemical cross-linking is not all controllable.	[1,78,79,80,81]

**Table 3:** CHs employed for different biomedical applications.

Major Biomedical Applications	Conductive Hydrogel Polymer	Conductive Hydrogel System	Specific Biomedical Applications	Year	Refs
Regenerative medicine	Polyethylene glycol (PEG)	miRNA/PGPC polyplex encapsulated in PEG hydrogels (miRNA/PGPC@PEG HG)	Intervertebral disc tissue engineering	2018	[109]
	Carboxymethyl chitosan (CMCH) Poly(3,4-ethylenedioxythiophene) (PEDOT)	Conductive hydrogels (PEDOT/CMCH)	Neural tissue engineering	2018	[110]
	Polyacrylamide (PAM)	Conducting polymer hydrogel (CPH) based on copolymerized PANI and PAM (PAM/PANI CPH)	Neural tissue engineering	2020	[111]
	Hyaluronic acid (HA)	HA and Pluronic F-127 (HA-F)	Corneal tissue engineering	2017	[112]
	Chitosan (CH)	Hemicellulose xylan/CH composite	Osseous tissue engineering	2016	[113]
	Chitosan (CH)	CH/CG composites	Cartilaginous tissue engineering	2018	[114]
Artificial organs	Polypyrrole (PPY)	Multiwalled carbon nanotubes (CNT) Beta-cyclodextrin (beta-CD) N-isopropylacrylamide (NIPAM)	Artificial heart	2018	[98]
	Polyacrylic acid (PAA)	Electrically conducting hydrogel nanocomposite based on silver nanoparticles–polyacrylic acid (PAA)	Artificial muscles	2014	[16]
Biosensors	Polyaniline (PANI)	Acid-templated polyaniline (PANI) Poly (ethylene glycol diglycidyl ether)	Glucose biosensor	2007	[57]
	Poly(acrylamide-co-lauryl methacrylate) (P(AAM-co-LMA))	Hybrid latex nanoparticles (HLPs) crosslinked P(AAM-co-LMA)	Motion/respiration biosensor	2019	[115]
	Polyacrylamide (PAAM)	Bio-conjugated polyacrylamide-based hydrogel (HBPAAM hydrogel)	Hepatitis B core antigen biosensor	2020	[116]
	Chitosan (CH)	PAAM–CH–PPy	Wearable biosensor	2018	[117]
	N-(9-fluorenylmethoxycarbonyl)-L, L-diphenylalanine (Fmoc-FF)	Peptide hydrogels encapsulating leishmania antigen(N-(9-fluorenylmethoxycarbonyl)-L, L-diphenylalanine (Fmoc-FF) encapsulating leishmania antigen)	Antigen biosensor	2017	[118]
Drug delivery systems	Alginate (Alg.)	Ionic crosslinked alginate hydrogels (calcium alginate hydrogels)	Drug delivery systems for the treatment of tumors	2014	[119]
	Polyethylene Glycol (PEG)	Sericin/hydrogel scaffold	Controlled drug delivery system	2019	[120]
	Poly(N-isopropylacrylamide) (PNIPAAM)	Poly(diketopyrrolopyrrole-alt-3,4-ethylenedioxythiophene)-PNIPAAM	Near-infrared light-controlled drug delivery system	2017	[121]
	Phenylboronic acid-grafted γ-Polyglutamic acid (PBA-PGA)	KGM/PBA-PGA	Insulin (Ins) with liraglutide (Lir) delivery system	2021	[122]
	Poly(acrylamidoglycolic acid) (PAGA)	Poly(acrylamidoglycolic acid) based nanocomposite (PAGA-NC)	Diclofenac sodium (DCF) delivery system	2017	[123]
Wound dressings	Methylacrylate gelatin (GelMA)	GelMA-PDA-ASP nanocomposite hydrogels	Dermal tissue engineering	2021	[124]
	Chitosan (CH)	Silver sulfadiazine loaded CH/PVP	Dermal tissue engineering	2019	[125]

## Data Availability

Data are available within the article.
